# Star Excursion Balance Test in Young Athletes with Back Pain

**DOI:** 10.3390/sports4030044

**Published:** 2016-08-23

**Authors:** Edem Korkor Appiah-Dwomoh, Steffen Müller, Miralem Hadzic, Frank Mayer

**Affiliations:** Clinical Exercise Science, Faculty of Health Science, University Outpatient Clinic, Potsdam 14469, Brandenburg, Germany; stefmue@uni-potsdam.de (S.M.); mhadzic@uni-potsdam.de (M.H.); fmayer@uni-potsdam.de (F.M.)

**Keywords:** young athletes, back pain, star excursion balance test

## Abstract

The Star Excursion Balance Test (SEBT) is effective in measuring dynamic postural control (DPC). This research aimed to determine whether DPC measured by the SEBT in young athletes (YA) with back pain (BP) is different from those without BP (NBP). 53 BP YA and 53 NBP YA matched for age, height, weight, training years, training sessions/week and training minutes/session were studied. Participants performed 4 practice trials after which 3 measurements in the anterior, posteromedial and posterolateral SEBT reach directions were recorded. Normalized reach distance was analyzed using the mean of all 3 measurements. There was no statistical significant difference (*p* > 0.05) between the reach distance of BP (87.2 ± 5.3, 82.4 ± 8.2, 78.7 ± 8.1) and NBP (87.8 ± 5.6, 82.4 ± 8.0, 80.0 ± 8.8) in the anterior, posteromedial and posterolateral directions respectively. DPC in YA with BP, as assessed by the SEBT, was not different from NBP YA.

## 1. Introduction

Back pain, particularly occurring in the lumbar region, has been considered rare in children and adolescents [1]. However, the most recent studies suggest it is a problem not only in children and adolescents [2,3], but also in young athletes (YA) [4,5,6,7,8]. Back pain causes a disruption of postural control [9] and can alter trunk muscle activity in chronic [10,11] and acute [12] patients. Therefore, there is the need for periodic assessment and monitoring, so as to identify and appropriately rehabilitate the impaired posture and alteration in trunk muscle activity.

To evaluate initial deficits resulting from injury, risk of injury and improvement after the intervention for an injury, postural control assessments are used. This can be carried out statically or dynamically depending on the task performed. In the general population, Ruhe et al. [13], using static measures, confirmed increased postural instability in individuals with low back pain compared to healthy controls. In athletes, however, static assessments are unable to challenge the postural control system and fail to obtain useful information [14]. Hence, there is a need for dynamic assessment. This form of assessment should involve some level of movement around a base of support and closely replicates the demands of physical activity in sports participation [15].

In assessing dynamic postural control (DPC), one test that has captured the attention of researchers and clinicians is the Star Excursion Balance Test (SEBT). Here, DPC is determined by how far a participant can reach while maintaining a base of support [16]. It is a simple, inexpensive, reliable tool that does not require special equipment and is effective in measuring multi-planar excursion and postural control [15]. The premise of this test is to determine if, while standing on an injured or affected limb to maintain stability, a deficit is produced in the reaching distances, indicating a deficiency in DPC that might be associated with the pathologic condition in the stance limb [15]. 

In people with a history of back pain (BP), there is a delay in the feed-forward postural response leaving the spine unprotected when movement of the lower limb occurs [17]. As such, one can assume that the vulnerability of the spine to further injuries would limit how far the limb would move in a dynamic test, such as the SEBT. As BP influences the trunk as well as lower limb movement [18], there is the possibility of detecting deficit in DPC using the measure of reach distance. In the only published article on the use of the SEBT to measure dynamic posture among low back pain subjects in the general population, Ganesh et al. [19] concluded that it is an effective tool to identify and measure reach deficits in this group of patients. Therefore, application of this tool in young athletes may prove a more challenging task that could help further assess and monitor DPC deficits in YA with BP. 

To the best of our knowledge, there is currently no published literature investigating dynamic posture in YA with BP using the SEBT. Therefore, this study aimed to determine if DPC, measured by SEBT reach distance, in YA with BP is different than NBP YA. The hypothesis was that there is a difference in the reach distance in BP and NBP YAs as measured by the SEBT.

## 2. Materials and Methods

### 2.1. Subjects 

A total of 53 YA with BP (14.7 ± 1.2 years, 62.4 ± 13.6 kg, 171.9 ± 10.5 cm, 6.1 ± 2.7 training years, 7.8 ± 3.3 training session/week, 108.5 ± 30.2 training minutes/session) and 53 YA NBP (14.5 ± 1.2 years, 59.6 ± 10.8 kg, 170.5 ± 10.5 cm, 5.7 ± 2.7 training years, 7.8 ± 3.3 training sessions/week, 102.3 ± 28.8 min/session) participated in the study. The athletes were from 13 different sports (boxing, athletics, rifle shooting, rowing, canoeing, judo, football, handball, cycling, wrestling, horse riding, gymnastics and volleyball). The 53 BP subjects were matched with 53 NBP according to age, height, weight, training years, training sessions/week and training minutes/session but not sports discipline. Subjects were recruited at a health check for YA applying to or already in an elite school of sports in Brandenburg, Germany. A pain questionnaire consisting of a numeric rating scale of 1 (no pain) to 5 (most severe pain) in the form of smiley faces was used to allocate participants into control or BP groups [20]. BP was not confined to a specific back region. Subjects with lower and upper limb injuries, head injuries, vision problems and any other complaints that could have affected balance measurement were excluded. Medical examination was carried out for all subjects before testing. The institution’s ethics committee gave ethical approval and the parent or guardian of each participant gave written informed consent before data collection.

### 2.2. Test Description

The SEBT is a measure of dynamic balance. The original version is composed of 8 lines extending 45° from the center of a grid made with an adhesive tape on the floor. Hertel et al. [21] reported redundancy of some of the directions and proposed a shorter version, which was used in this study. The shortened version includes the anterior, posteromedial and posterolateral directions. Excellent interrater reliability with normalized and non-normalized reach distance scores [22], as well as strong intra-rater reliability (ICC = 0.84–0.87) and test–retest reliability (ICC = 0.89–0.93) [23], have been reported. There is also literature to support the construct and predictive validity of the SEBT [16,21], although no gold standard exists for measuring dynamic balance. 

### 2.3. Measurement Procedure

**Figure 1:**
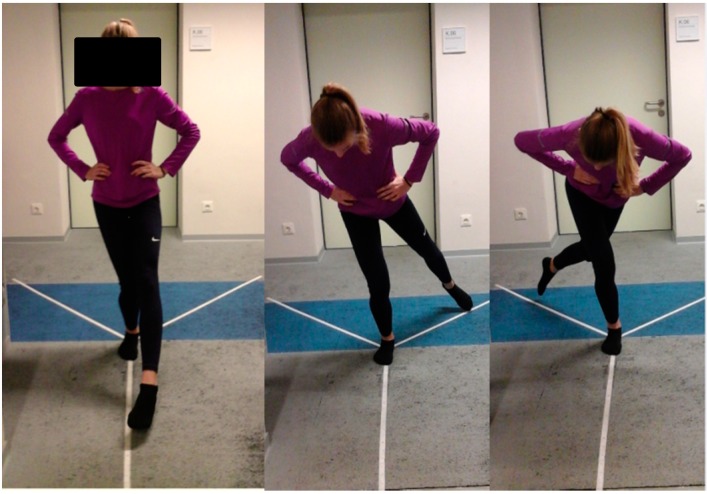
Testing situation: Star Excursion Balance Test in the anterior, posterolateral and posteromedial directions, respectively.

Age, gender, weight, height, number of training years, training days per week, training minutes per session and type of sports engaged in by subjects were recorded. Oral instructions, as well as a demonstration of how the test should be performed, were given to the participants. The SEBT directions were constructed by affixing 3 tape measures with a centimeter scale on the laboratory floor. The first reach direction was aligned anterior to the apex; the other two were oriented 135° to the first in posteromedial and posterolateral directions [24]. The order of the starting limb was randomized and the chronology of the directions was defined (1. Anterior; 2. Posteromedial; 3. Posterolateral). The subject’s starting foot is placed at the convergence of the reach directional lines of the SEBT [24]. In the process the lateral malleolus is positioned at the intersection point of the 3 directions with the foot’s longitudinal axis oriented at the anterior direction. The starting position is a bilateral stand. Subjects stood with socks while keeping their hands on their hips. Maintaining a single leg stance, they were instructed to reach out as far as possible with the non-stance limb along the marked tape, point to the most distal portion with their great toe and return the limb back to the starting position [25]. Subjects practiced each direction 4 times before the main test in order to minimize learning effect [23,26]. This was followed by the recording of 3 successful trials in each direction for both legs, always with a 10-s rest between each test [21]. The limb length of subjects was then taken with a measuring tape. This was defined as the distance from the anterosuperior iliac spine to the medial malleolus [27]. Maximum reach distance was visually read by the same examiner for all subjects. The examiner is a final year doctoral student with 5 years working experience as a physiotherapist and 4 additional years of working with young athletes as part of the doctoral studies. Prior to testing the examiner received training at the University of Potsdam Outpatient Clinic, a licensed medical examination center of the German Olympic Sports federation. A trial was considered invalid if the reaching foot did not return to the starting position, touched down while reaching out, the support limb shifted, the heel of the support foot did not stay in contact with the ground or if the hands were removed from the hips (Figure 1). 

### 2.4. Outcome Measure

Outcome measures of interest were the mean normalized reach distance of the 3 trials, and a composite reach distance score (CRDS) [28]. For normalization, the mean reach distance of the 3 trials was divided by limb length (cm) and multiplied by 100 for a percentage score. The composite reach distance was calculated using the sum of the 3 normalized reach distances divided by 3 times the limb length, multiplied by 100 [28].

### 2.5. Data and Statistical Analysis

Relevant data for analysis was handwritten into a case report form after which computation was performed. Data was first descriptively analyzed (mean ± standard deviation), followed by independent *t*-tests, paired *t*-tests and Mann-Whitney U tests for normally and non-normally distributed data, respectively. Post hoc power analysis was carried out using G*Power 3.1.9.2 [29] to determine whether the research was adequately powered. Effect size was calculated using the formula (mean of BP group—mean of NBP group)/pooled standard deviation of both group. SPSS version 22 (SPSS Inc., Chicago, IL, USA) was used for analysis. Significance was set at α = 0.05.

## 3. Results

Normalized reach distances for BP and NBP groups in each direction and composite reach distance scores (CRDS) for right lower limb (RLL) and left lower limb (LLL), are presented in Table 1 and Table 2. There was no significant difference between the RLL and LLL of BP and NBP subjects for the reach distances and the CRDS. 

**Figure 2:**
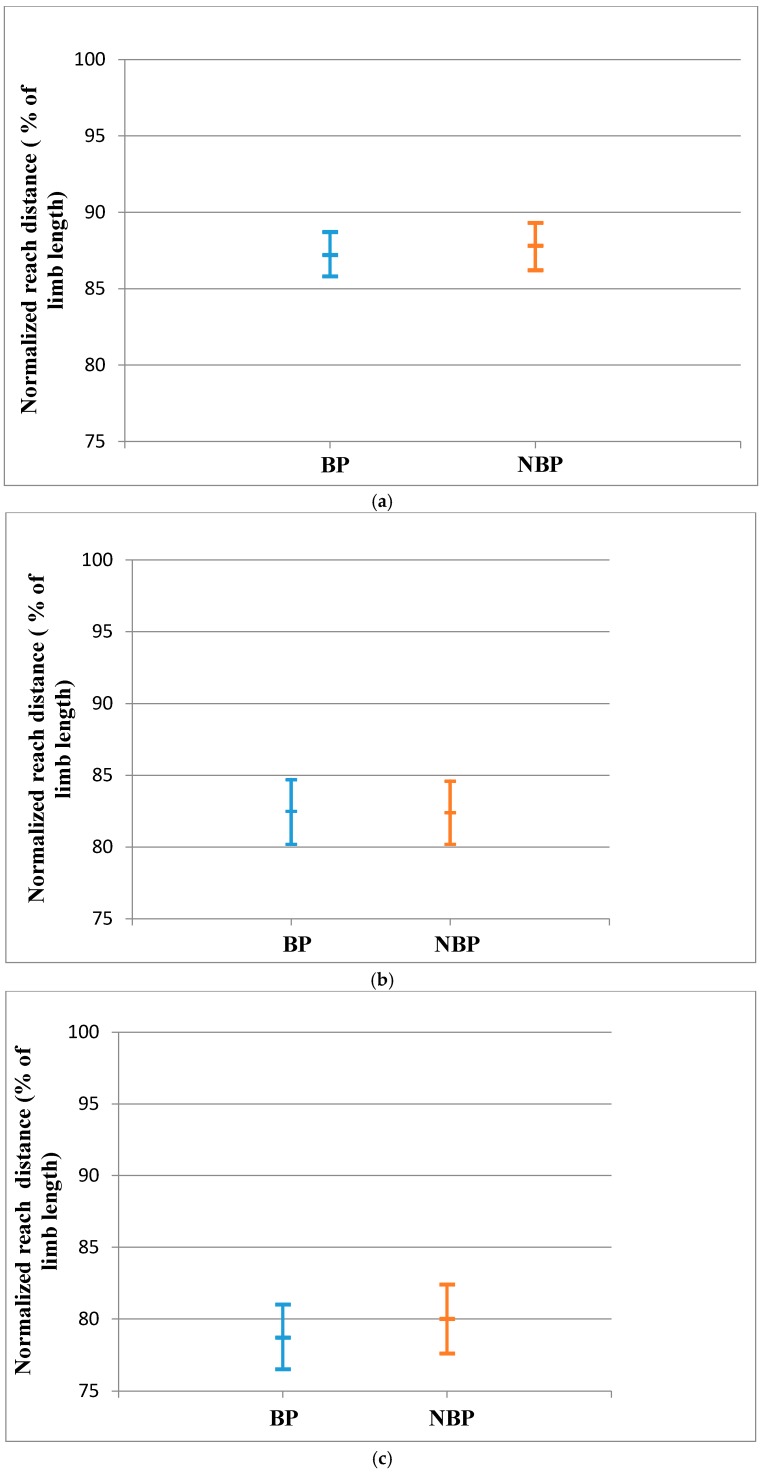

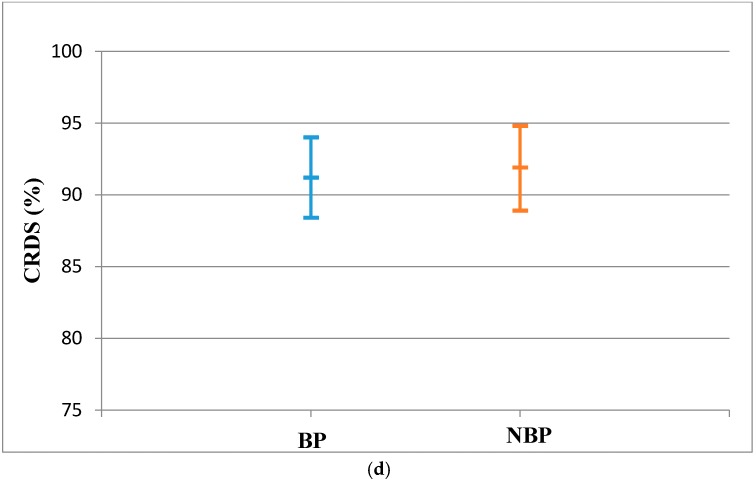
(**a**) Anterior Reach Distance for RLL (Mean and CI 95%); (**b**) Posteromedial reach distance for RLL (Mean and CI 95%); (**c**) Posterolateral reach distance for the RLL (Mean and CI 95%); (**d**) CRDS for the RLL (Mean and CI 95%).

There was no statistically significant difference between BP and NBP subjects for the RLL in all directions of the SEBT. The effect sizes for the reach distances were small (Table 3). The population means of the RLL for the BP and NBP young athletes did not show significant evidence of a difference as observed from the 95% confidence interval (Figure 2a–d).

**Figure 3:**
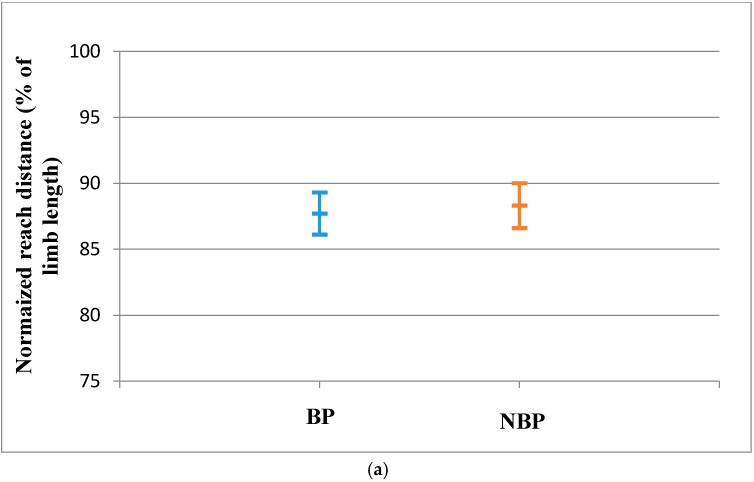

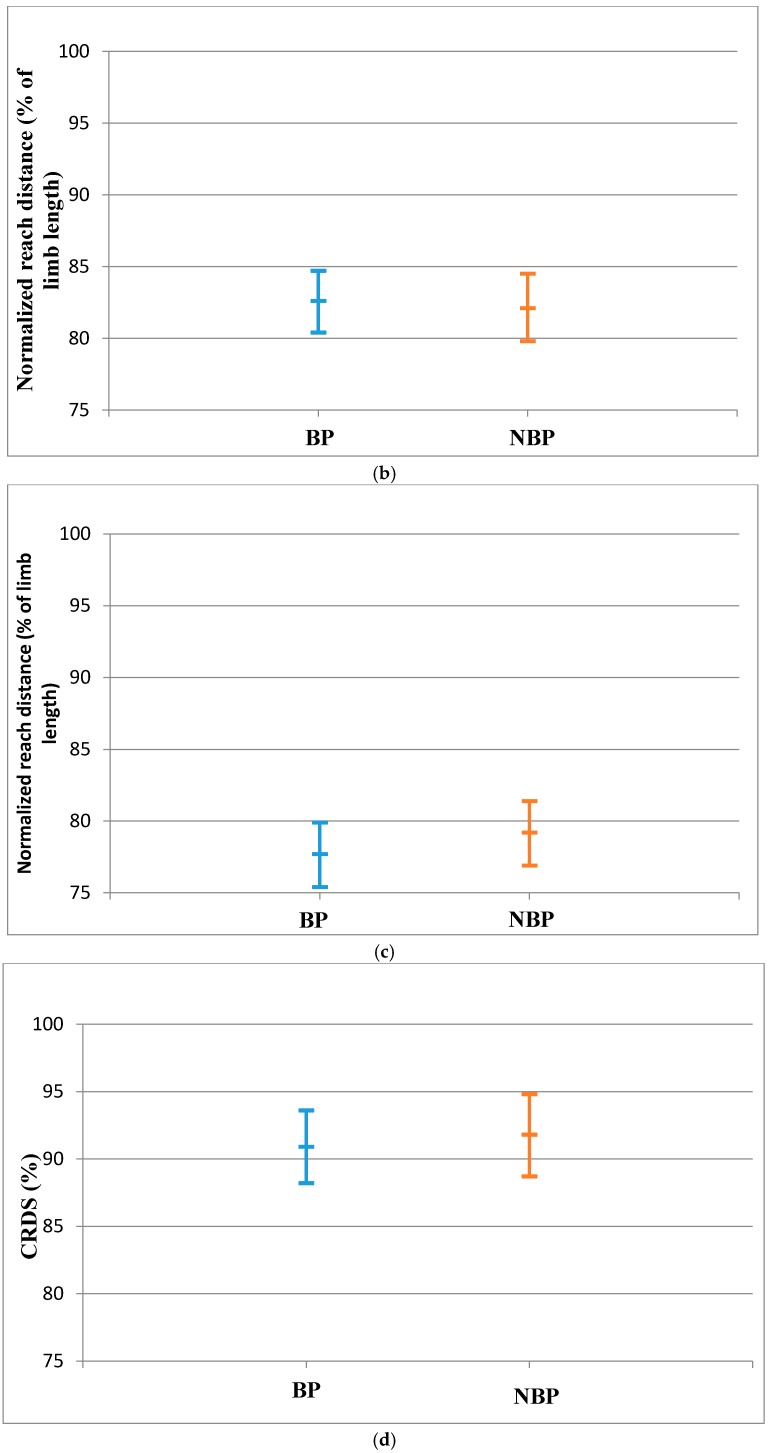
(**a**) Anterior Reach Distance for LLL (Mean and CI 95%); (**b**) Posteromedial reach distance for LLL (Mean and CI 95%); (**c**) Posterolateral reach distance for the RLL (Mean and CI 95%); (**d**) CRDS for the LLL (Mean and CI 95%).

The reach distances and CRDS of the LLL for BP were not statistically significantly different from the NBP subjects. The effect sizes for the reach distances were also small (Table 4). There was also no significant evidence that the population means of the LLL for the BP and NBP young athletes are different as shown by the 95% confidence interval (Figure 3a–d). 

## 4. Discussion

This study aimed at determining if dynamic postural control, as measured by normalized SEBT reach distances, differed between YA with and without BP. The human postural system operates on the basis of integrated information from three independent sensory sources: visual, vestibular and somatosensory [30]. In order for the body to maintain balance it relies on rapid, continuous feedback from these sensory sources to execute smooth and coordinated neuromuscular actions [31]. Therefore, damage to sensory tissues and pain inhibition in the lumbar spine and trunk, as occurs in BP, are believed to affect the postural control mechanism [10,32]. This leads to the adoption of alternative postural control strategies in order to cope with the new demands introduced by pain [10]. This could be why the BP subjects performed as well as the controls in all directions. It could also be that athletes generally have superior balance ability due to sport participation [33] masking the effects of pain on balance. In addition to this, the visual system provides the body with visual cues for use as reference points in orienting the body in space. It also provides feedback from the reach leg during movement and allows observation of scored reach distances [24]. This also could have contributed to the present result in which the BP YA reached as far as their matched controls. In a dynamic task such as the SEBT, visual and vestibular inputs are important and tend to be the most reliable [34]. However, a reduction in vision feedback [34] that should occur during the posteromedial and posterolateral reaches still did not alter reach distances between the two groups of interest. According to Jacobs et al. [35], there is reduced proprioceptive feedback from mechanoreceptors of the trunk and hip joint as a result of altered sensory input at the site of BP. Hence one would have thought this would lead to a differentiation between the BP subjects and their matched controls. The current results could thus be a confirmation that athletes are indeed more skilled at focusing and attending to important sensory cues, when producing refined motor responses [36], like those during the SEBT. 

The only published literature on SEBT in relation to BP is that of Ganesh et al. [19]. Unlike the present study, they showed a statistically significant difference between low back pain and control subjects. Their back pain subjects reported lower anterior (72.61 ± 6.91), posteromedial (74.19 ± 8.50) and posterolateral (63.19 ± 1.18) reach distances compared to the present study, 87.2 ± 5.3, 82.5 ± 8.2 and 78.7 ± 8.1 for the same directions respectively. The control subjects of Ganesh et al. [19] also recorded lower anterior (82.38 ± 5.11) and posterolateral (76.30 ± 9.32) but higher posteromedial (83.06 ± 1.02) reach distances compared to the current study of 87.8 ± 5.6, 82.4 ± 8.0 and 80.0 ± 8.6 for the anterior, posteromedial and posterolateral reach distances, respectively. This result adds to the knowledge that athletes generally have superior balance ability compared to the general population [33]. Our results, however, cannot be compared directly to the above mentioned study due to different study populations, severity of BP and limb tested. Young athletes (age range 12–18) were measured in this study, while Ganesh et al. [19] measured non-athletes with an age range of 22–50 years.

There was no significant difference between the right and left lower limbs of young athletes with and without back pain on all the reach distances of the SEBT and the CRDS. The current result, though it confirms the findings of Holden et al. [28] and Alonso et al. [37], cannot be directly compared. The aforementioned investigations worked on the dominant and non-dominant limbs of athletes (13 ± 0.3 years) and non-athletes (26 ± 5.0 years) respectively while the current one did not take limb dominance into consideration. In interpreting the above results, gender was not taken into consideration. There is lack of agreement on the effect of gender on the SEBT, with literature reporting no effects [28,38,39] and significant effects [40,41] after normalization. Hence further investigation is required in order to ascertain the effect of gender on the current results.

The 95% confidence intervals for all the reach distances and CRDS for both BP and NBP subjects include the null hypothesis means and also showed large overlaps. Hence the conclusion can be drawn that there is no statistically significant difference between the reach distances of BP and NBP young athletes for both RLL and LLL. Finally, because of the sample size (N = 53), limited statistical power may have played a role in limiting the significance of the reach distances between the BP and NBP groups. Power analysis showed that on the basis of the lowest effect size observed in the present study (d = 0.01), approximately 123,652 subjects would be needed in both BP and NBP groups to obtain statistical power at level 0.80 [42]. Therefore, this supports the results showing no differences. 

### Limitations of the Study 

The pain questionnaire may be considered a limitation of this study as it only assessed pain within the 7 days prior to participation in the study. Hence, the possibility of varying phases and location of BP and its effect on the current results cannot be ruled out. In addition to this, a mean pain score of 2.6 for our cohort might be too low to produce differences. Another limitation might be the varying sports disciplines considered together in the study. It would have been interesting to focus on the major sports discipline presented by our athletes, as according to Thorpe & Ebersole [43], the SEBT may be sensitive to specific sport related adaptations. Also, distinct skill requirements and environmental demands of different sports likely pose different challenges to the sensorimotor systems [38]. Accordingly, each sport will likely require different levels of sensorimotor processes to perform skills as well as protect the neuromuscular system from injury [38]. Consequently, any differences that might have been present could have been masked due to the various sports disciplines involved in this study.

## 5. Conclusions

YA with and without BP do not differ in reach distance as measured on the SEBT. Hence, deficits in dynamic postural control, as a result of BP, could not be assessed using the SEBT reach distance. Therefore, our hypothesis is rejected. This may imply that the SEBT, although a simple tool, is not effective in discriminating between YA with and without BP. Future studies should use questionnaires that assess BP longer than the previous 7 days and consider specific BP classification. Investigations comparing young athletes from different sports disciplines would be interesting.

## Figures and Tables

**Table 1:** Normalized Reach Distance, Composite Reach Distance Score (CRDS) and limb length (cm) (mean ± sd) for BP subjects.

Normalized Reach Distance (Limb Length %) for BP Subjects			
	RLL	LLL	*p* Value
Anterior	87.2 ± 5.3	87.7 ± 5.8	0.27
Posteromedial	82.5 ± 8.2	82.6 ± 7.9	0.86
Posterolateral	78.7 ± 8.1	77.7 ± 8.0	0.06
CRDS	91.2 ± 10.1	90.9 ± 9.8	0.53
Limb length (cm)	91.3 ± 6.3	91.4 ± 6.3	0.37

**Table 2:** Normalized Reach Distance, CRDS and limb length (cm) (mean ± sd) for NBP subjects.

Normalized Reach Distance (% Limb Length) for NBP Subjects			
	RLL	LLL	*p* Value
Anterior	87.8 ± 5.6	88.3 ± 6.2	0.22
Posteromedial	82.4 ± 8.0	82.1 ± 8.6	0.53
Posterolateral	80.0 ± 8.8	79.2 ± 8.2	0.19
CRDS	91.9 ± 10.8	91.8 ± 11.1	0.81
Limb length (cm)	91.3 ± 6.4	91.3 ± 6.5	0.63

**Table 3:** Normalized Reach Distance (% limb length), CRDS (mean ± sd) and effect size for the RLL of subjects.

Normalized Reach Distance (% of Limb Length) for RLL				Effect Size
	BP	NBP	*p* Value	
Anterior	87.2 ± 5.3	87.8 ± 5.6	0.63	0.11
Posteromedial	82.5 ± 8.2	82.4 ± 8.0	0.65	0.01
Posterolateral	78.7 ± 8.1	80.0 ± 8.6	0.44	0.16
CRDS	91.2 ± 10.1	91.9 ± 10.8	0.75	0.07

**Table 4:** Normalized Reach Distance (% of limb length), Composite Reach Distance Score (CRDS) and effect size for the LLL of subjects.

Normalized Reach Distance (% of Limb Length) for LLL				Effect Size
	BP	NBP	*p* Value	
Anterior	87.7 ± 5.8	88.3 ± 6.2	0.61	0.10
Posteromedial	82.6 ± 7.9	82.1 ± 8.6	0.79	0.06
Posterolateral	77.7 ± 8.0	79.2 ± 8.2	0.41	0.25
CRDS	90.9 ± 9.8	91.8 ± 11.1	0.68	0.09
